# Future Leader to Watch – Olayemi Joseph Olajide

**DOI:** 10.1242/bio.058548

**Published:** 2021-01-25

## Abstract

Future Leader to Watch is a series of interviews with the first authors of a selection of papers published in Biology Open, helping early-career researchers promote themselves alongside their papers. Olayemi Joseph Olajide is first author on ‘Molecular mechanisms of neurodegeneration in the entorhinal cortex that underlie its selective vulnerability during the pathogenesis of Alzheimer's disease’, published in BiO. He is a Research Fellow in the Center for Studies in Behavioral Neurobiology, Department of Psychology, Concordia University, Montreal, Quebec, Canada, investigating the mechanisms of molecular neurodegeneration during the pathogenesis of Alzheimer's disease.


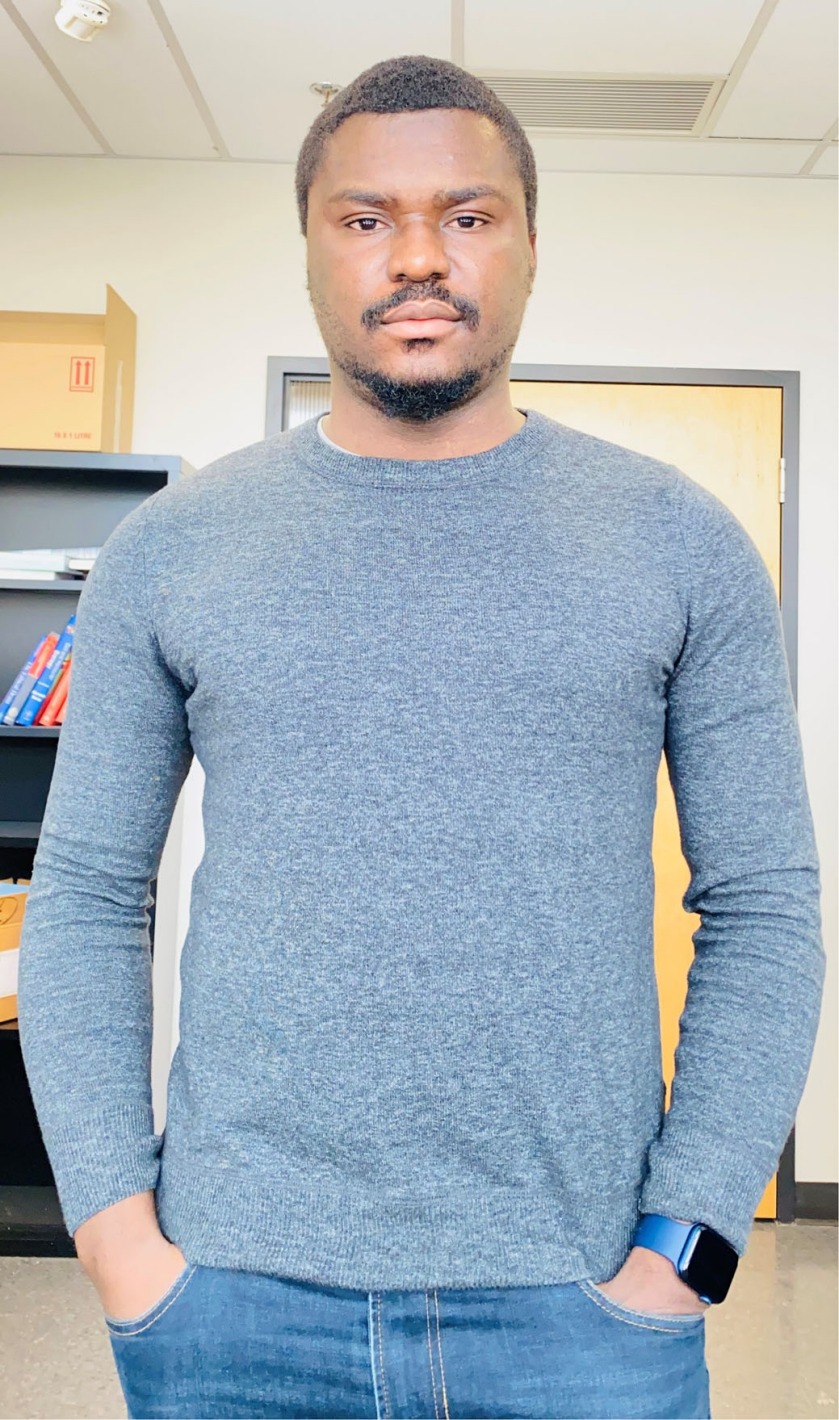


**Olayemi Joseph Olajide**

**What is your scientific background and the story of how you got to where you are today?**

I trained as an anatomist and neurobiologist, focusing on mechanisms of neurodegeneration during Alzheimer's disease and the application of target-based therapeutic strategies. I had my PhD training at the University of Ilorin in Nigeria, where I am originally from, but I did a good part of my doctoral research in the laboratory of Prof. Stephen Price at the University College London in England. I then worked for about two years as a postdoctoral fellow at the International Centre for Genetic Engineering and Biotechnology in Italy, before joining the laboratory of Prof. Andrew Chapman as a research fellow at the Centre for Studies in Behavioural Neurobiology, Concordia University, Canada. I also hold the position of a Lecturer in the Department of Anatomy, University of Ilorin, Nigeria. Basically, my research goal is to better understand the processes that lead to cognitive decline during Alzheimer's disease-related neuropathology and how the disease progression can be halted. I am currently studying how amyloid beta peptide impact synaptic functions in the entorhinal cortex using electrophysiological and molecular biology techniques, which is relevant to the understanding of basic mechanisms of memory loss during Alzheimer's disease. I find this topic exciting because there is surprisingly a lot of new things to learn.

“… surprisingly few studies have focused on understanding why the entorhinal cortex may be particularly vulnerable to molecular neurodegeneration.”

**What is the most important take-home message of your review?**

The entorhinal cortex contributes vitally to cognitive processes and memory formation, and despite growing indication that degeneration of neurons within this medial temporal lobe structure may play a central role in the early memory loss that is associated with Alzheimer's disease, surprisingly few studies have focused on understanding why the entorhinal cortex may be particularly vulnerable to molecular neurodegeneration. In our review, we critically assessed the unique pathways within the entorhinal cortex that may explain its selective susceptibility to early degeneration during Alzheimer's disease. Based on the intrinsic properties of the entorhinal cortex and the current findings of our lab, we projected on how the dysfunction of specific molecular signals may perturb entorhinal functions and how this may contribute to Alzheimer's-related cognitive decline. We concluded that a thorough understanding of the mechanisms underlying molecular neurodegeneration in the entorhinal cortex is crucial to unraveling how Alzheimer's disease develops, and for the advancement of potent therapeutic strategies.

**What has surprised you the most while researching this review?**

Although substantial progress has been made in the studying of Alzheimer's disease, it is astonishing how very little research has been done to understand the mechanisms of molecular neurodegeneration in the entorhinal cortex and how this contributes to early loss of neurons during Alzheimer's disease. What is more interesting is that the progressive nature of the pathological changes that occur in the entorhinal cortex can serve as effective biomarkers that may aid prediction and diagnosis of Alzheimer's disease long before its clinical manifestation, which is a crucial objective in Alzheimer's research. The belief is that pre-symptomatic intervention will be far more potent than applying therapy after the onset of disease, and a robust understanding of processes mediating the early changes in the entorhinal cortex may serve as an important landmark in Alzheimer's disease research.

“… pathological changes that occur in the entorhinal cortex can serve as effective biomarkers that may aid prediction and diagnosis of Alzheimer's disease long before its clinical manifestation …”

**What do you feel is the most important question that needs to be answered to move the field forward?**

It is not yet clear how proteins such as amyloid beta peptide, that are ubiquitously expressed, accumulate and spread selectively in vulnerable neurons, but not in neighbouring cell types and brain regions. Understanding the basis of this selective neuronal or regional vulnerability is key to gaining insights into the molecular underpinnings of neurodegenerative diseases like Alzheimer's disease.

There is also lack of specific markers to fully identify the unique morphologic and functional properties of different brain regions, but the development of such markers is expected to allow investigations to better determine the roles of distinct cell types in regional susceptibility to neurodegeneration.

**What changes do you think could improve the professional lives of early-career researchers?**

The most important factors are for early-career researchers to have more support from their mentors and to work on topics that truly interest them. Young researchers should be given more allowance to make mistakes without being harshly judged or subjected to confidence-damaging remarks. Further, mentors and professional bodies should institute more career development initiatives that educate young scientists on the different career paths that they could pursue after their training, other than remaining in the academia. Finally, I think early-career researchers, particularly PhD students and postdocs, are grossly underpaid in most parts of the world. This often leave them with too many economical/social uncertainties and insecurities, which can disrupt the succinct process of learning and novel discoveries.

**What's next for you?**

In the interim, I am enjoying the pursuit of new discoveries related to the mechanisms underlying molecular neurodegeneration in the entorhinal cortex during Alzheimer's-type pathology, as well as teaching undergraduate level neurobiology at Concordia University. I am also focusing on grant applications that can further support the maintenance of my own research program wherein students can gain practical research mentoring and laboratory training.
